# Mapping the two distinct proliferative bursts early in T‐cell development

**DOI:** 10.1111/imcb.12670

**Published:** 2023-07-19

**Authors:** Seungyoul Oh, Dhruti Parikh, Jiyao Xiao, Xin Liu, Karen Gu, Mark MW Chong

**Affiliations:** ^1^ St Vincent's Institute of Medical Research Fitzroy VIC Australia; ^2^ Department of Medicine (St Vincent's) University of Melbourne Fitzroy VIC Australia; ^3^ Faculty of Science University of Melbourne Parkville VIC Australia

**Keywords:** Cell cycle, proliferation, single‐cell RNA sequencing, T‐cell development

## Abstract

T‐cell development occurs in the thymus and is tightly regulated to produce a diverse enough repertoire of mature T cells that can recognize any potential pathogen. The development of T cells is dependent on small numbers of uncommitted precursors that continually seed the thymus from the bone marrow. As they progress along the developmental pathway, there is a massive expansion in cell number to generate the necessary diversity in T‐cell receptor chain usage. It is recognized that there are two proliferative bursts that occur early in T‐cell development, one prior to β‐selection and one after, and these are responsible for the expansion. While the proliferation following β‐selection is well‐characterized, the earlier proliferative burst has yet to be precisely defined. In this study, we employ single‐cell RNA sequencing coupled to trajectory inference methods to pinpoint when in T‐cell development thymocytes are induced into cell cycle. We show that the first proliferative burst is initiated in the double‐negative (DN) 2a stage before T lineage commitment occurs, with cell cycling downregulated by the DN3a stage. A second burst is then initiated at the DN3b stage, immediately after β‐selection. We subsequently employ fluorescence‐activated cell sorting–based analysis for DNA content to confirm these two proliferative bursts.

## Graphical abstract

We employed single‐cell RNA sequencing to map the proliferative bursts early in the T‐cell development. Double‐negative 2a (DN2a) and DN3b were identified as the two points when developing thymocytes were induced into cell cycle.

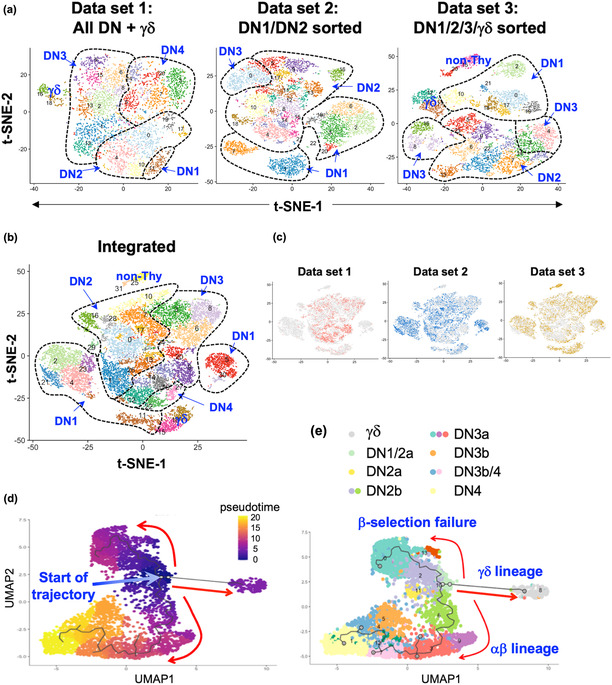

## INTRODUCTION

T‐cell development is dependent on the continuous seeding of multipotent progenitors from the bone marrow. The earliest stages are termed as double negative (DN) because the thymocytes lack CD4 and CD8 expression. In mice, these are subdivided into four consecutive stages (DN1 to 4) based on CD44 and CD25 expression.^1^ It is still not entirely clear what the immediate downstream populations of the thymic seeding progenitors are, but studies suggest that it is a c‐KIT (CD117)‐expressing DN1 subpopulation that maintains the greatest multipotency and thus likely to be the earliest thymic progenitor (ETP). These cells maintain the capacity to differentiate into myeloid, B and natural killer cell lineages, in addition to T cells.^2^ From the DN1/ETP stage, thymocytes progress through the DN2, DN3 and DN4 stages and then upregulate CD4 and CD8 to become CD4^+^CD8^+^ double positive. Most mature T‐cell lineages are selected from double positive–staged thymocytes. However, γδ T cells develop from the DN1 and DN2 stages.^3^, ^4^

Two key developmental checkpoints occur during the DN stages. The first is T lineage commitment at DN2, when multipotency is lost and differentiation into a T‐cell is irreversibly fixed.^2^ Pre‐ and post‐checkpoint DN2 cells are designated DN2a and DN2b, respectively. DN2a cells still maintain the ability to differentiate into natural killer cell and myeloid lineages.^5^ Expression of GATA3, TCF1 and HES1, critical specification factors for the T lineage program, are all upregulated by this point.^2^ Expression of BCL11B is then required to complete T‐cell specification in DN2b cells.^6^

The second developmental checkpoint, β‐selection, occurs at DN3, the purpose of which is to select cells that have successfully rearranged the *Tcrb* gene. Selection of cells that express a pre‐T‐cell receptor (TCR; TCRβ complexed with pre‐Tα) verifies that these precursors have undergone productive *Tcrb* gene rearrangements before progression is permitted.^7^ Pre‐ and post‐β‐selection DN3 thymocytes are designated as DN3a and DN3b, respectively. Developing γδ T cells are thought to be selected on the complete TCRγδ heterodimeric complex.^7^

Proliferation is integral to the development of thymocytes. ETPs constitute 0.01% of cells in the thymus, DN3 thymocytes 1%, while up to 85% of thymocytes are double positive.^8^ This almost 10^4^‐fold expansion is required to generate TCR diversity and is thought to involve two rounds of proliferation (Supplementary figure 1). The initial round of proliferation occurs prior to β‐selection to increase the precursor pool for rearrangement of the *Tcrb* locus and evidence suggests that Notch and cytokine signaling are involved.^9^, ^10^ A second burst occurs in DN3b and DN4 thymocytes, following β‐selection. Entry into cell cycle is critical for the progression to the double positive stage and is required to increase the pool of TCRβ‐expressing thymocytes that can rearrange the *Tcra* locus.^11^

Of the two proliferative bursts, only the later burst has been extensively characterized. β‐selection is a definitive checkpoint where only thymocytes that successfully recombine the *Tcrb* gene and express a functional TCRβ polypeptide can continue to develop. The initiation of proliferation entirely coincides with pre‐TCR signaling.^12^ Thus, the connection of this checkpoint with the triggering of proliferation could be clearly defined. However, the proliferation that occurs prior to β‐selection remains less‐well characterized. Recent advances in single‐cell RNA sequencing (scRNA‐seq) allows us to probe developmental pathways at a resolution not previously possible. In this study, we employ a series of DN scRNA‐seq data sets to pinpoint the proliferative bursts that occur early in T‐cell development and then validate these with FACS‐based DNA quantification.

## RESULTS AND DISCUSSION

### Inferring a developmental trajectory from scRNA‐seq data sets of DN thymocytes

An increasing number of studies have shown that gene expression analysis of scRNA‐seq data can be employed to infer the cell cycling state of cells based on the expression levels of cell cycle genes.^13^ Furthermore, we can order cells along a developmental trajectory in pseudotime to infer precisely when cell cycling is initiated.

**Figure 1:**
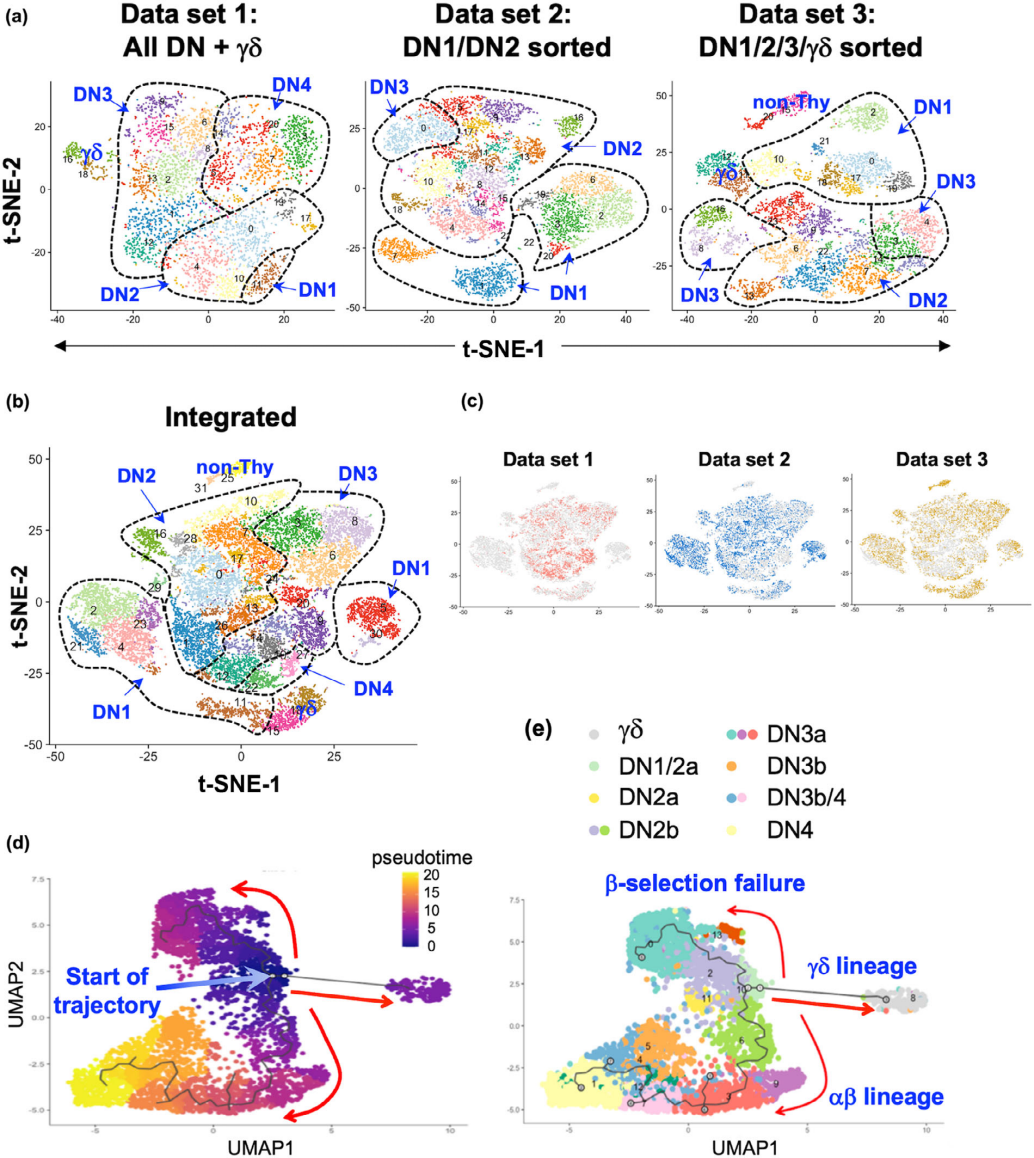
Clustering and trajectory analysis of DN thymocyte scRNA‐seq data. **(a)** Three independent data sets of mouse DN thymocytes were analyzed: (1) total DN and TCRγδ^+^ cells, (2) only DN1 and DN2 cells and (3) sorted populations of DN1/DN2, DN3 and TCRγδ^+^ cells that were mixed back together after sorting at a ratio of 55% to 30% to 15%, respectively. See Supplementary figure 2 for the sorting strategy. Following processing of the 10× data in Cell Ranger, doublets were removed with DoubletFinder and cell cycle genes were regressed out. **(b)** The integration of the three data sets with Seurat using anchors following preprocessing and batch corrections implemented with SCTransform. Cell cycle genes were also regressed out. Clustering of each independent data set or the integrated data set was performed at a resolution of 2.0, which resulted in DN1, DN2, DN3 and DN4 thymocytes separating into distinct clusters, based on the expression of the marker genes indicated in Supplementary figure 3. Indicated are the clusters that corresponded to the broad DN1/2/3/4 stages in T‐cell development, TCRγδ^+^ cells or non‐thymocytes. **(c)** Contribution of each independent data set to the integrated data set. **(d)** Developmental trajectory inferred with Monocle 3. The individual cells are colored coded by position in pseudotime. **(e)** Color coding of the Monocle 3 trajectory by DN stage. DN, double negative; scRNA‐seq, single‐cell RNA sequencing; TCR, T‐cell receptor; Thy, thymocytes; t‐SNE, t‐distributed stochastic neighbor embedding.

We recently published three 10× scRNA‐seq data sets of DN thymocytes.^3^ The first consisted of total DN and TCRγδ^+^ thymocytes, the second data set consisted only of DN1 and DN2 thymocytes and the third data set was generated from DN1/DN2, DN3 and TCRγδ^+^ thymocytes sorted separately and then mixed back together after sorting (Figure 1 and Supplementary figure 2). These sort strategies were employed to ensure that enough DN1 and DN2 thymocytes could be captured.

Following removal of low‐quality cells and doublets (with DoubletFinder^14^), 4256 cells were obtained for data set 1, 8151 cells for data set 2 and 6846 cells for data set 3. We analyzed each data set separately (Figure 1a) as well as integrating the three together (Figure 1b, c). Cell cycle genes were regressed out and then clustering was performed at a resolution of 2.0, which was required to separate DN1, DN2, DN3 and DN4 cells into distinct clusters, based on the expression of key marker genes (Supplementary figures 1 and 3).

We next assembled developmental trajectories of the data using different trajectory inference algorithms. Data set 1 (total DN cells) was assessed with Monocle 2^15^ (Supplementary figure 4a), while the combined data set was assessed with Monocle 3^16^ (Figure 1d, e) and Slingshot^17^ (Supplementary figure 4b). As previously shown with Monocle 2 and Slingshot,^3^ and now with Monocle 3, three branches starting from DN1 thymocytes were predicted. One corresponding to the αβ developmental pathway, ending with DN4 cells; one corresponding to the γδ pathway, developing directly from DN1 cells; and one terminating with DN3 cells that had failed β‐selection, which express genes associated with apoptosis and cellular senescence.^3^

### Gene expression analysis suggest that thymocytes upregulate cell cycling at DN2a and again at DN3b

**Figure 2:**
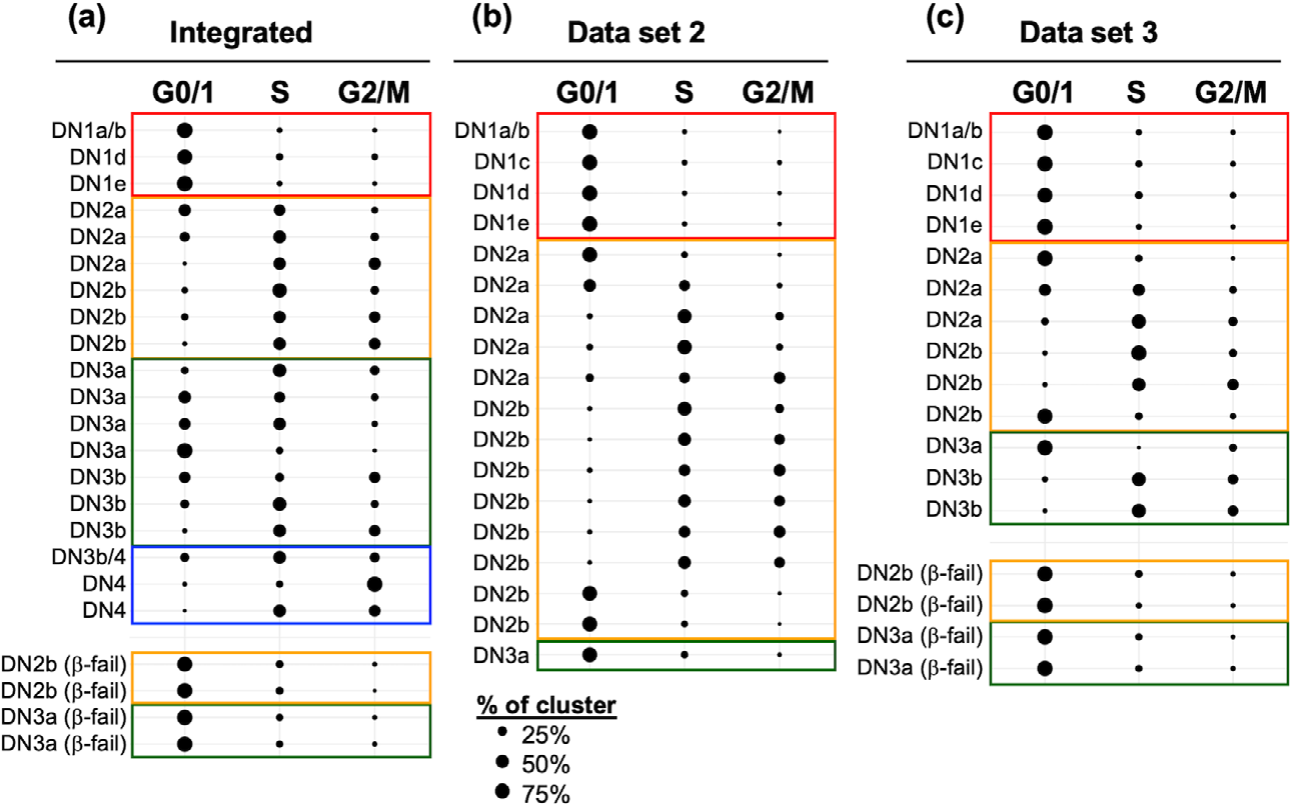
Mapping the two proliferative bursts during early T‐cell development using scRNA‐seq data. **(a)** The integrated data set, **(b)** data set 2 (DN1 + DN2 thymocytes) and **(c)** data set 3 (DN1/DN2, DN3 and γδ thymocytes mixed after sorting) were first clustered. Data sets 2 and 3 were analyzed individually to achieve a greater resolution of the DN2 stage. The assignment of cells within each cluster to cell cycle phase was then performed in Seurat. Each line indicates the percentage of cells assigned to G1/0, S or G2/M phase within an individual cluster. The clusters within each analysis are ordered based on the trajectories inferred by Monocle 3 in Figure 1d and Slingshot in Supplementary figure 4b. Clusters that correspond to β‐selection failure are shown separately. DN, double negative; scRNA‐seq, single‐cell RNA sequencing.

The ordering of DN thymocytes along the developmental trajectories was then used as a reference against which the various clusters of DN thymocytes were ordered. The cells within each cluster were then assigned to S or G2/M if expressing genes associated with those cell cycle phases.^13^ Cells not expressing S or G2/M genes were considered in the G0/1 phase. The percentage of cells in each phase was calculated for each cluster. Assessment of the integrated data set inferred that cell cycling is upregulated at the DN2a stage, with all DN2a and DN2b clusters exhibiting higher frequencies of cycling cells compared with the DN1 clusters (Figure 2a). Cell cycling was downregulated in DN3a clusters but then upregulated again in DN3b and DN4 clusters. The second proliferative burst clearly coincides with β‐selection, as expected. However, the first proliferative burst did not coincide with the other important early checkpoint, T lineage commitment and instead appears to occur prior, at least by this scRNA‐seq analysis.

We next assessed data sets 2 and 3 separately to gain a higher resolution of DN2 cells (Figure 2b, c). These individual data sets again inferred upregulation of cell cycling at DN2a instead of DN2b.

### FACS analysis confirms that proliferation is upregulated at DN2a

Next, we sought to experimentally confirm the upregulation of proliferation at the DN2a stage. To do this, we employed FACS‐based analysis to identify thymocytes at specific developmental stages based on the expression of previously defined cell surface markers (Supplementary figure 5). Inclusion of 4′,6‐diamidino‐2‐phenylindole for DNA content then allows for the quantification of cells in the G1/0, S or G2/M phases. Following gating out of lineage marker‐expressing cells, the broad DN1/2/3/4 thymocyte stages were subdivided based on CD44 *versus* CD25 expression. CD24 *versus* CD117 then delineated DN1 thymocytes into the a/b/c/d/e subpopulations as defined by the Petrie laboratory,^18^ with DN1a/b corresponding to the ETPs. CD117^high^ *versus* CD117^low^ delineated DN2a *versus* DN2b cells as defined by the Rothenberg laboratory^5^ and we recently showed that simultaneous inclusion of CD90 achieves better resolution.^3^

**Figure 3:**
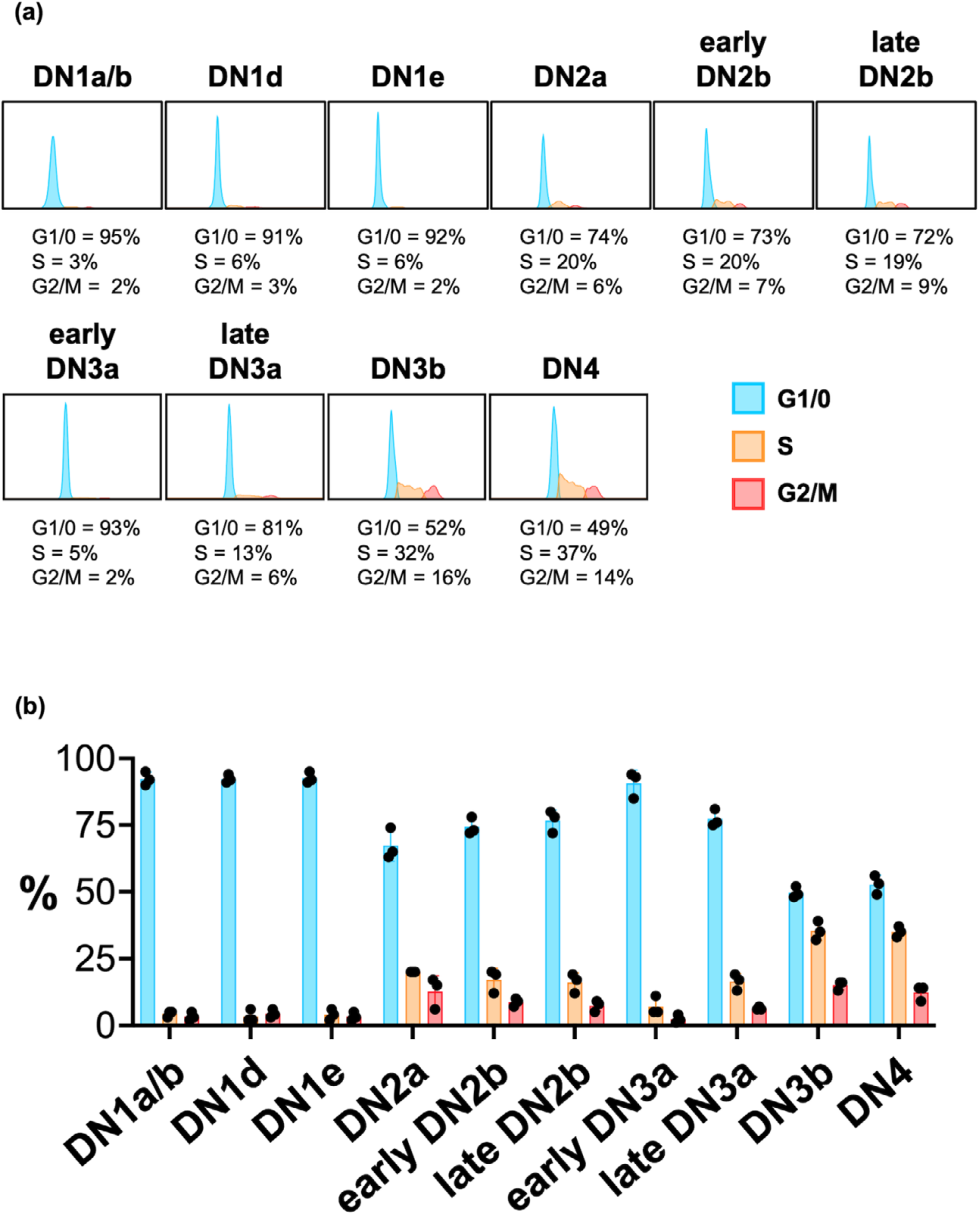
FACS analysis for DNA content confirms that thymocytes cell cycling is upregulated at the DN2a stage and again at the DN3b stage. DN thymocyte subpopulations were identified with the gating strategy shown in Supplementary figure 5 and DAPI staining for DNA content was employed to quantify cells in G1/0, S or G2/M phase. **(a)** An example of DNA content analysis of DN subpopulations. **(b)** The mean ± s.e.m. of three experiments, with each dot indicating an experimental datapoint. The cells from up to three mice were pooled for each experiment. DAPI, 4′,6‐diamidino‐2‐phenylindole; DN, double negative; FACS, fluorescence‐activated cell sorting.

Consistent with the scRNA‐seq analysis (Figure 2), all DN1 subpopulations exhibited minimal cell cycling, with only about 5% of cells in S or G2/M phases (Figure 3). Also consistent with the scRNA‐seq analysis, there was then a clear upregulation in cycling cells at DN2a, with one‐quarter of cells in S or G2/M phases. This is consistent with previous studies that compared the DNA content of total DN1, 2, 3 or 4 cells.^18^, ^19^ Although at that time these studies lacked knowledge of markers to resolve pre‐ and post‐T lineage committed cells, it was clear that there are more DN2 cells cycling than DN1 cells.

**Figure 4:**
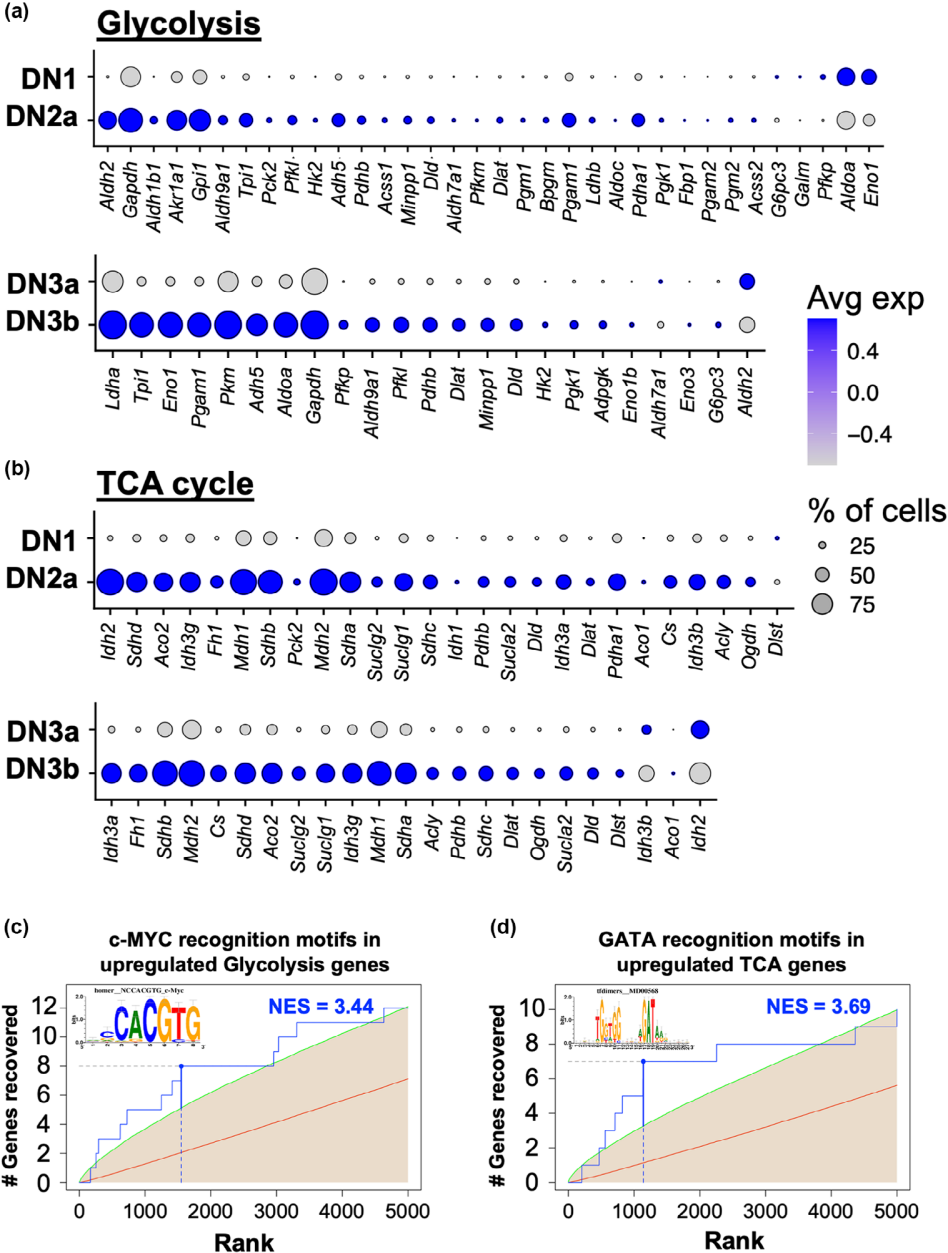
Upregulation in cell cycling at the DN2a and DN3b stages is associated with an increased expression of metabolic genes. The gene expression profiles of total DN1 *versus* DN2a, and DN3a *versus* DN3b were extracted from the scRNA‐seq data. Differential expression analysis was then performed for genes associated with **(a)** glycolysis (KEGG map 00010) or **(b)** TCA cycle (KEGG map 00020). Shown are only genes with significantly different expression (adjusted *P* < 0.05) between DN1 and DN2a or between DN3a and DN3b. The genes are ordered by largest to smallest average fold change. The genes found to be upregulated from the DN1 to DN2a stage were analyzed for transcription factor recognition motifs with RcisTarget. **(c)** An enrichment of c‐MYC recognition sites was identified in the upregulated glycolysis gene set, **(d)** while an enrichment of GATA recognition sites was identified in the upregulated TCA cycle gene. Shown are the recovery curves for each analysis, where the recovery of each specific transcription factor motif is indicated by the blue line. The average recovery of all motifs is indicated by the red line, while average recovery + standard deviation is indicated by the green line. Also shown are the NES for each analysis, which is calculated based on the AUC distribution for all motifs in the gene set. A score for > 3.0 is considered significant. AUC, area under the curve; DN, double negative; KEGG, Kyoto Encyclopedia of Genes and Genomes; NES, normalized enrichment score; scRNA‐seq, single‐cell RNA sequencing; TCA, tricarboxylic acid.

Compared with post‐β‐selection DN thymocytes, preselected thymocytes have been shown to be less metabolically active but a step up in metabolic status has also been observed between DN1 and DN2 thymocytes.^20^ This aligns with our finding that proliferation is upregulated between DN1/ETP and DN2a thymocytes. However, these previous studies only assessed total DN2 thymocytes. We therefore wanted to confirm that an upregulation in metabolism status occurs from the DN2a stage, when cell cycling first increases. Indeed, we found that DN2a cells upregulated the expression of numerous genes associated with glycolysis and the tricarboxylic acid cycle (Figure 4a, b).

The DN1/ETP to DN2a transition is not known to be associated with a checkpoint or extrinsic signal and therefore it is unclear what would initiate the entry of DN2a thymocytes into cell cycle. The transcription factors that establish T‐cell identity, such as GATA3 and TCF1, are already highly expressed at this point.^2^ It may be concurrent expression of multiple pioneering factors, including these, that is responsible for initiating proliferation prior to the T lineage commitment checkpoint. Of these, GATA3 has been shown to drive accessibility of the N‐Me *Myc* gene enhancer,^21^ and c‐MYC is thought to play a role in thymocyte proliferation.^22^ Interestingly, we found that the upregulated glycolysis gene set (between DN1 and DN2a) is enriched for c‐MYC recognition motifs (Figure 4c), while the upregulated tricarboxylic acid cycle gene set is enriched for GATA recognition motifs (Figure 4d). Whether the GATA3–c‐MYC interaction is indeed responsible for triggering an upregulation in proliferation and metabolic gene expression at DN2a will need to be experimentally validated.

### FACS analysis confirms proliferation is again upregulated following β‐selection

We also confirmed by FACS‐based analysis for DNA content that the second burst occurs following β‐selection. For this, total CD44^−^ DN thymocytes were subdivided into early DN3a, late DN2a, DN3b and DN4 based on CD28 *versus* CD25 expression as previously described.^23^ We confirmed that the CD25^lo^CD28^+^ gate corresponds to true DN3b stage cells (Supplementary figure 5e, f). Consistent with the scRNA‐seq analysis there was a clear upregulation in the frequency of cycling cells at the DN3b stage, with half of cells in the S or G2/M phases as determined by DNA content (Figure 3). Further, this was maintained into the DN4 stage. There was also a further increase in metabolism as inferred by the upregulation of genes associated with glycolysis and the tricarboxylic acid cycle between the DN3a and DN3b stages (Figure 4). This is consistent with previous metabolic profiling of DN thymocytes.^20^ Thus, our data confirm that the second proliferative burst does indeed correlate with β‐selection.

Unlike the earlier proliferative burst, this latter burst has been extensively characterized. Although studies suggested that entry into cell cycle may not be dependent on β‐selection itself, it is now clear that pre‐TCR signaling induces cyclin D3 expression *via* NFAT and LCK, and in the absence of cyclin D3, T‐cell development is impaired.^24^ Notch signaling is also required for initiating cell cycle entry following β‐selection. Notch induces the expression of the F‐box protein FBXL1, while pre‐TCR signaling induces expression of FBXL12.^25^ These are components of the SCF ubiquitin ligase complex that promote the degradation of CDKN1B, which is required for the proliferation of thymocytes that have passed β‐selection.^25^ Thus, Notch and pre‐TCR signaling are thought to work cooperatively to induce this latter proliferative burst.

By using scRNA‐seq and FACS analysis, we have thus precisely mapped the two proliferative bursts early in T‐cell development. However, while the β‐selection–associated burst is well defined, further studies are required to elucidate what is responsible for initiating the first proliferative burst.

## METHODS

### scRNA‐seq data sets and data processing

Three previously generated 10× Genomics scRNA‐seq data sets of mouse thymocytes (Gene Expression Omnibus repository under accession number GSE188913)^3^ were analyzed for this study. The data sets were of (1) total DN and TCRγδ thymocytes; (2) DN1 and DN2 thymocytes only and (3) DN1/DN2 thymocytes, DN3 thymocytes and TCRγδ thymocytes that were sorted separately and then mixed back together at a ratio of 55% to 30% to 15%, respectively. For each experiment, the cells from four C57BL/6 mice at 6 or 7 weeks of age were pooled for the sorting.

The sequencing files were demultiplexed and aligned to the mm10 *Mus musculus* transcriptome reference, and count matrices were extracted using Cell Ranger Single Cell software (version 4.0.0, 10× Genomics). The outputs were preprocessed with Seurat (version 4.3.0).^26^ Low‐quality cells with > 5000 or < 1000 detected genes (nFeature_RNA) and > 12.5% mitochondrial gene expression were removed, and DoubletFinder (version 2.0.3) was applied to remove likely sequencing doublets before downstream analyses.^14^ Highly variable genes were identified by the variance‐stabilizing transformation method and selected for unsupervised linear principal component analysis. After regressing out cell cycle genes, unsupervised clustering was implemented using the minimal number of components to explain > 90% of variance of gene expression.

The three data sets were also integrated for analyses. Each data set was preprocessed and batch corrections were implemented with SCTransform (version 0.3.5).^27^ Integration anchors were then identified between the data sets for merging using canonical correlation analysis and cell cycle gene regression in Seurat.^26^ Unsupervised clustering was again implemented using the minimal number of components to explain > 90% of variance of gene expression.

### Pseudotime trajectory inference

Pseudotime trajectories were inferred from the DN thymocyte data sets with Monocle 3 (version 1.0.0).^16^ Preprocessing steps (e.g. normalization, estimation of size factor) were performed with the function *preprocess_cds()* in the package. A further round of dimensional reduction was applied using *reduce_dimension()* with reduction_method = “UMAP” and preprocess_method = “PCA”. In order to align with the UMAP output from Seurat, the cell embeddings that were calculated in the last step were replaced by cell embeddings calculated by RunUMAP from Seurat. The functions *cluster_cells()* and *learn_graph()* were applied to the data set to infer trajectories.

### Assignment of cell cycle states from scRNA‐seq data

Calculation of cell cycle scores for individual cells within the 10× data sets was performed in Seurat using the function CellCycleScoring.^13^ The scoring is based on the expression level of a previously determined list of cell cycle markers. Genes associated with the S and G2/M phases were stored with cc.genes in Seurat. Cells were then classified into either G2/M, S or G1/0 phase using these scores. Cells that did not have high expression of S genes or G2/M genes were assigned to G1/0 phase.

### Mice and analysis of thymocytes by FACS

Thymuses were harvested from C57BL/6 mice at 6 or 7 weeks of age. All experiments were approved by the St Vincent's Hospital Animal Ethics Committee and performed under the Australian code for the care and use of animals for scientific purposes.

Total thymocytes were obtained by crushing the thymus through a metal sieve to generate a single‐cell suspension. The cells were washed with phosphate‐buffered saline (PBS; Sigma‐Aldrich, St Louis, MO, USA) and filtered through a 70‐μm sieve to remove any clumps. For each experiment, the cells of up to three mice were pooled and then CD4‐ and CD8‐expressing cells were depleted using anti‐CD4 and CD8 magnetic‐activated cell sorting beads (Miltenyi Biotec), according to the manufacturer's instructions.

For DNA content analysis, the resulting CD4/8‐depleted cells were stained with antibodies against cell surface markers. See Supplementary table 1 for the list of antibodies used. All antibodies were purchased from Thermo Fisher. The cells were then washed with cold PBS and fixed with 0.5% paraformaldehyde (Sigma‐Aldrich) in PBS for 15 min on ice. Cells were washed two times with cold PBS and resuspended in PBS containing 1 μg mL^−1^ 4′,6‐diamidino‐2‐phenylindole (Thermo Fisher, Waltham, MA, USA), 200 μg mL^−1^ ribonuclease A (Sigma‐Aldrich) and 0.1% Triton X‐100 (Sigma‐Aldrich).

For intracellular TCRβ expression analysis, the cells were first stained with antibodies against cell surface markers, fixed and permeabilized with the FIX & PERM Cell Permeabilization Kit (Thermo Fisher), and then stained with an antibody against TCRβ. The cells were then resuspended in PBS + 0.5% bovine serum albumin without 4′,6‐diamidino‐2‐phenylindole.

All FACS data were acquired on an LSRFortessa III (BD Biosciences, Franklin Lakes, NJ, USA) and then analyzed with FlowJo software version 10.7.0 (BD Biosciences). Cell cycle analysis was performed with the “Cell Cycle” tool imbedded in FlowJo using the Watson Pragmatic model, where G0/1 and G2/M cells are assumed to be normally distributed and to fit a Gaussian curve.

### Differential gene expression analysis of metabolic genes

The differentially expressed genes were identified using the *FindAllMarkers* function in Seurat.^28^ The differentially expressed genes with an adjusted *P*‐value < 0.05, logfc.threshold = 0 and min.pct = 0 were selected and ranked by fold‐change. The differentially expressed genes for specific Kyoto Encyclopedia of Genes and Genomes (KEGG) pathways was identified using the R package KEGGREST (version 1.36.3).^29^

### Transcription factor recognition site analysis

The RcisTarget package^30^ in Bioconductor (version 3.8) was used to identify significantly enriched transcription factor recognition motifs within upregulated gene sets. The motif annotation database “motifAnnotations_mgi_v9” and the gene‐motif ranking database “mm10_10kbp_up_10kbp_down_full_tx_v10” were obtained from https://resources.aertslab.org/cistarget/. The motif enrichment analysis was performed using the *cisTarget()* function with default settings. The recovery curve was plotted using the *getSignificantGene()* function.

## AUTHOR CONTRIBUTIONS

**Seungyoul Oh:** Conceptualization; formal analysis; investigation; methodology; visualization; writing – original draft. **Dhruti Parikh:** Formal analysis; investigation; visualization; writing – original draft. **Jiyao Xiao:** Conceptualization; formal analysis; methodology; visualization; writing – original draft. **Xin Liu:** Data curation; formal analysis; methodology; visualization. **Karen Gu:** Data curation; formal analysis; investigation; visualization. **Mark MW Chong:** Conceptualization; funding acquisition; project administration; supervision; writing – original draft; writing – review and editing.

## CONFLICT OF INTEREST

The authors declare no conflicts of interest.

## Supporting information


Supplementary figures 1‐5
Supplementary table 1

## Data Availability

The scRNA‐seq data sets employed in this study are openly available in the Gene Expression Omnibus repository under accession number GSE188913.
