# Supplementary material for: Mapping the two distinct proliferative bursts early in T‐cell development
Source: Immunol Cell Biol. 2023 Jul 19;101(8):766–74. doi: 10.1111/imcb.12670 (PMC10952215; doi:10.1111/imcb.12670)
Supplement: Supplementary file 1 — Supplementary figures 1‐5 Supplementary table 1 [file IMCB-101-766-s001.pdf]

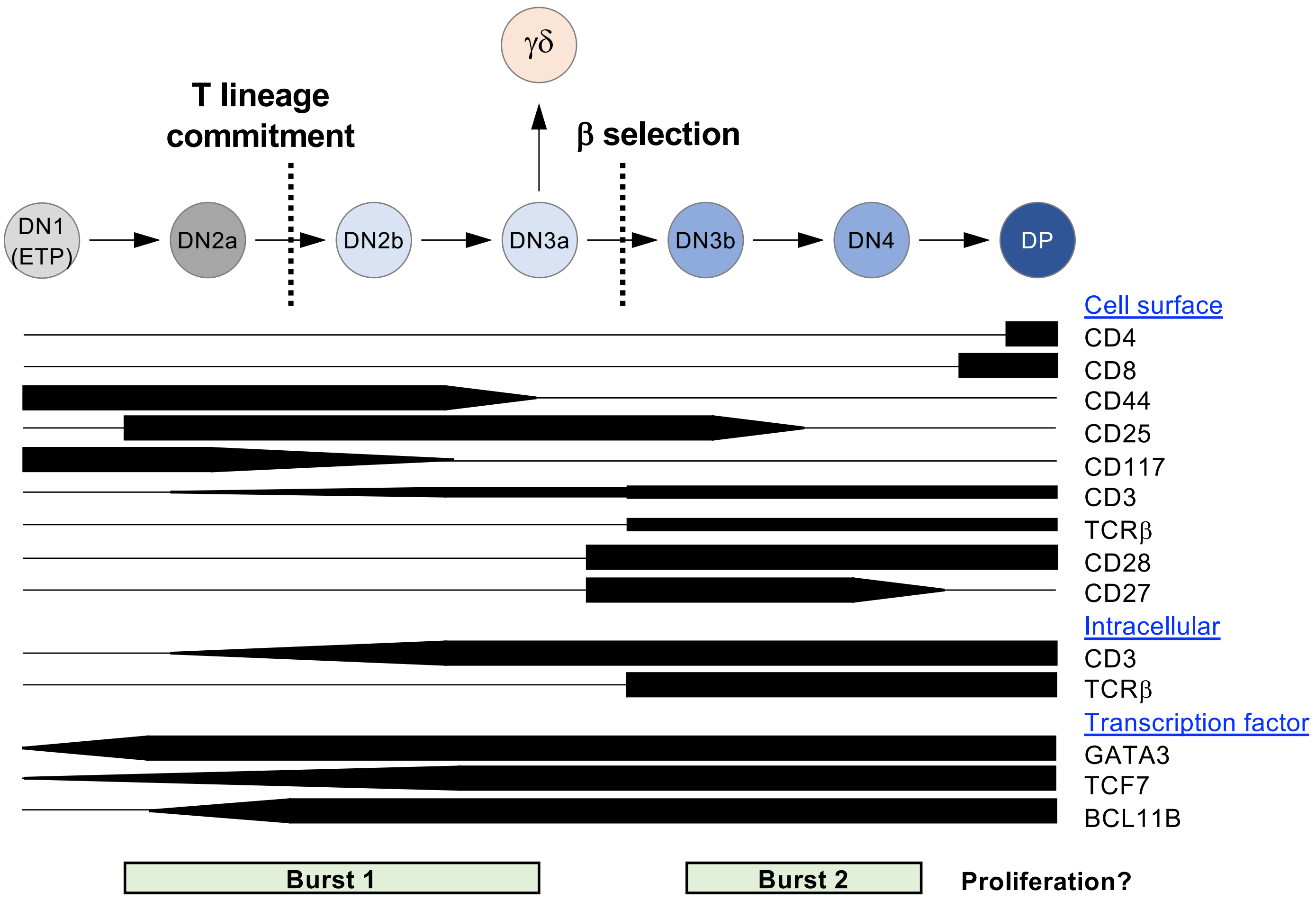

Supplementary figure 1: Summary of early mouse T cell development. The stages are referred to as double negative (DN) due to lack of CD4 or CD8 expression. Two key checkpoints are associated with these early stages: T lineage commitment and β-selection. The former demarcates DN2a from DN2b cells, and the later demarcates DN3a from DN3b cells. Indicated are previously defined cell surface markers and transcription factors used to identify these early developmental stages. Note, CD3 and TCRβ are primarily intracellular-localised at this point, with only low cell surface expression levels. Also indicated is when the proliferative bursts that expand thymocyte cell number are thought to occur.

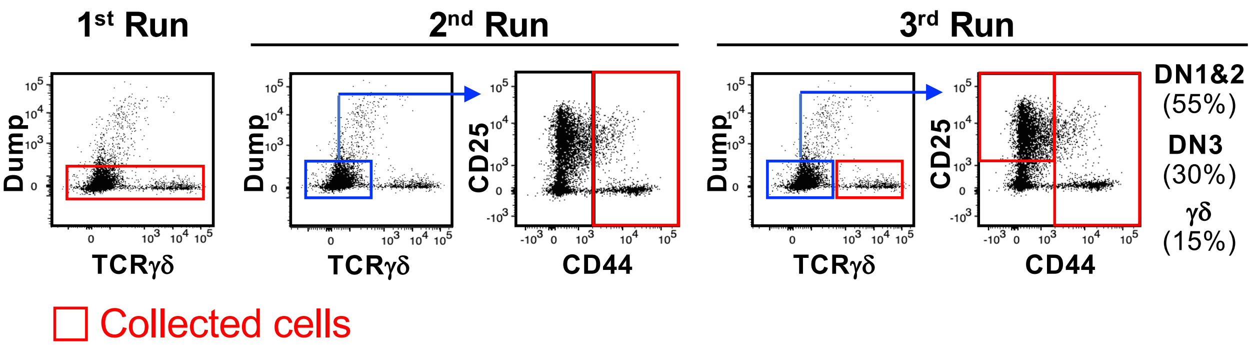

Supplementary figure 2: Summary of the sort strategy employed for capturing DN thymocytes for three 10x scRNAseq runs. These datasets were generated for a previously reported study [1]. The first run consisted of total DN and TCRγδ^{+} thymocytes, the second run consisted only of DN1 and DN2 thymocytes, and the third run consisted of sorted DN1/DN2, DN3 and TCRγδ^{+} thymocytes, which were then mixed back together at a ratio of 55% to 30% to 15% respectively. Dump = CD4, CD8, B220, CD11b, CD11c, NK1.1 and TCRβ.

[1] Oh S, Liu X, Tomei S, Luo M, Skinner JP, Berzins SP, Naik SH, Gray DHD, Chong MMW. Distinct subpopulations of DN1 thymocytes exhibit preferential γδ T lineage potential. *Front Immunol* 2023; 14:1106652.

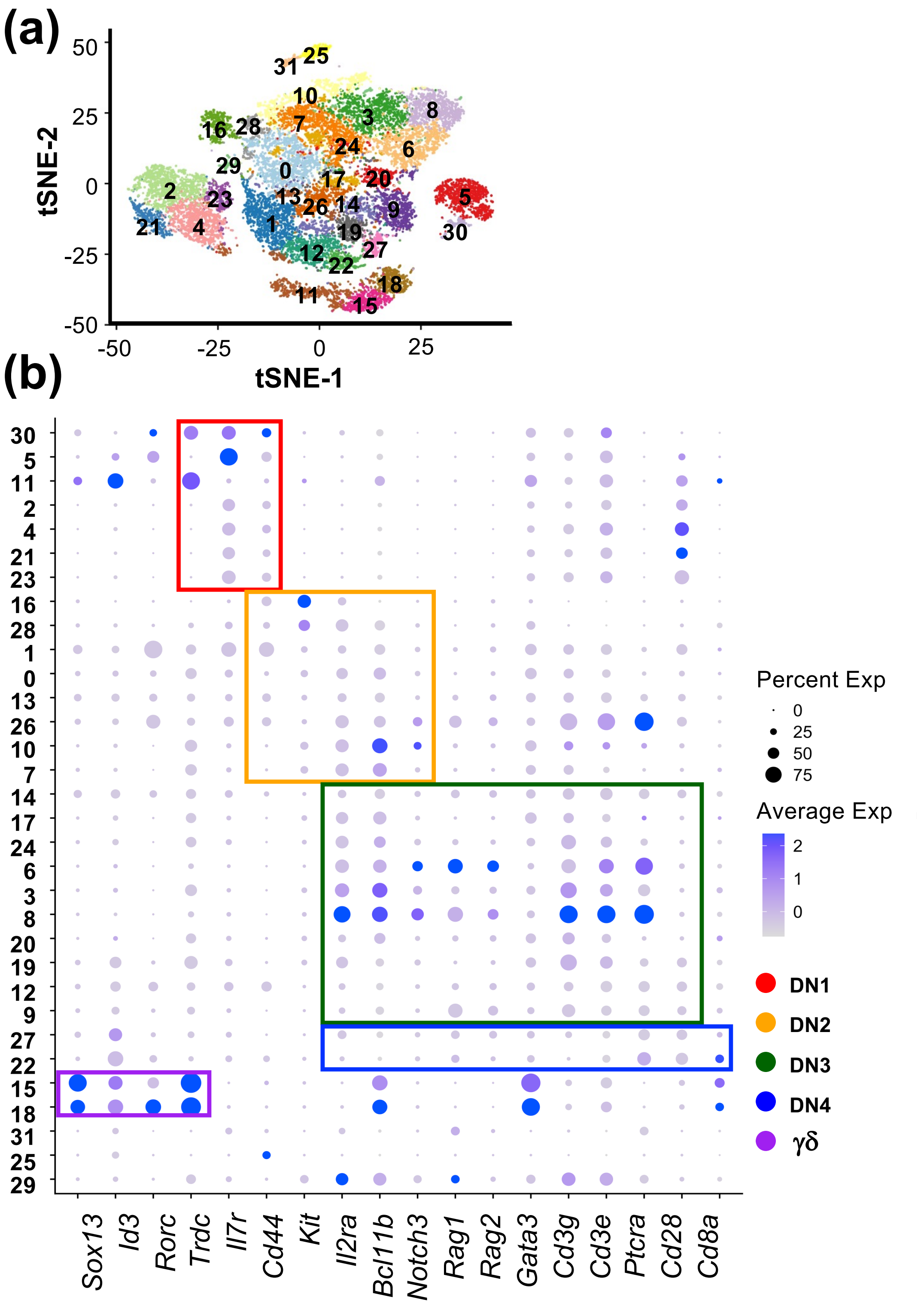

Supplementary figure 3: Assigning clusters to DN thymocyte stage. **(a)** Datasets 1, 2 & 3 were integrated with SCTransform, then clustered at resolution of 2.0, which was the minimum required to resolve DN1, DN2, DN3 and DN4 thymocytes into separate clusters. **(b)** Staging of clusters was based on expression of key marker genes, including those shown in the expression dot plot. Markers that particularly contribute to assignment are grouped in the coloured boxes.

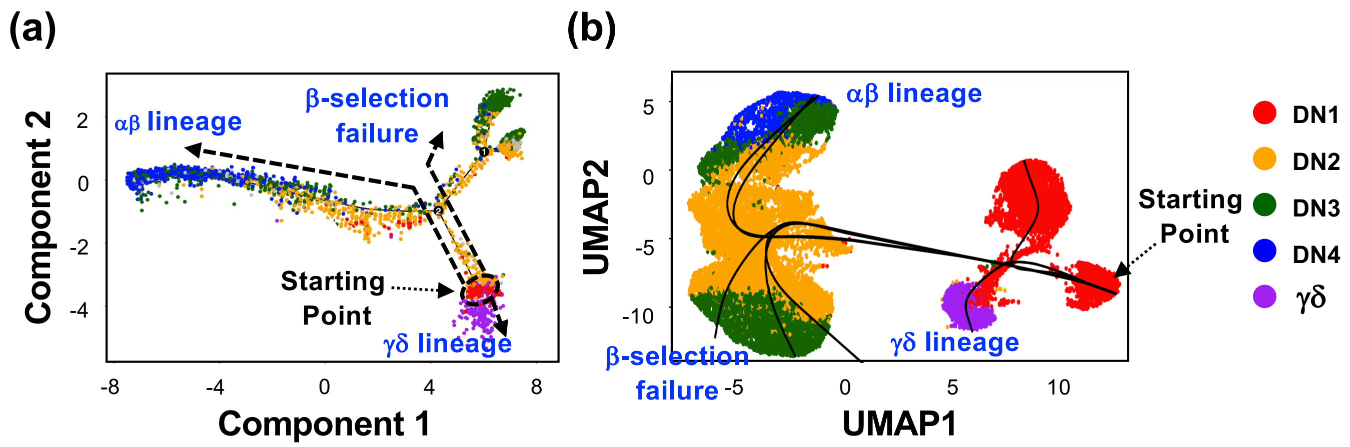

Supplementary figure 4: Inferring a developmental trajectory of DN and γδ thymocytes from scRNAseq data. **(a)** Dataset 1, consisting of sorted total DN + γδ thymocytes were analysed by Monocle 2. **(b)** Datasets 1, 2 & 3 were integrated, then analysed by Slingshot. Individual cells are coloured-coded to developmental stage based on expression of the marker genes described in Supplementary Figures 1 and 2. These trajectory analyses of the data were previously reported [1].

[1] Oh S, Liu X, Tomei S, Luo M, Skinner JP, Berzins SP, Naik SH, Gray DHD, Chong MMW. Distinct subpopulations of DN1 thymocytes exhibit preferential γδ T lineage potential. *Front Immunol* 2023; 14:1106652.

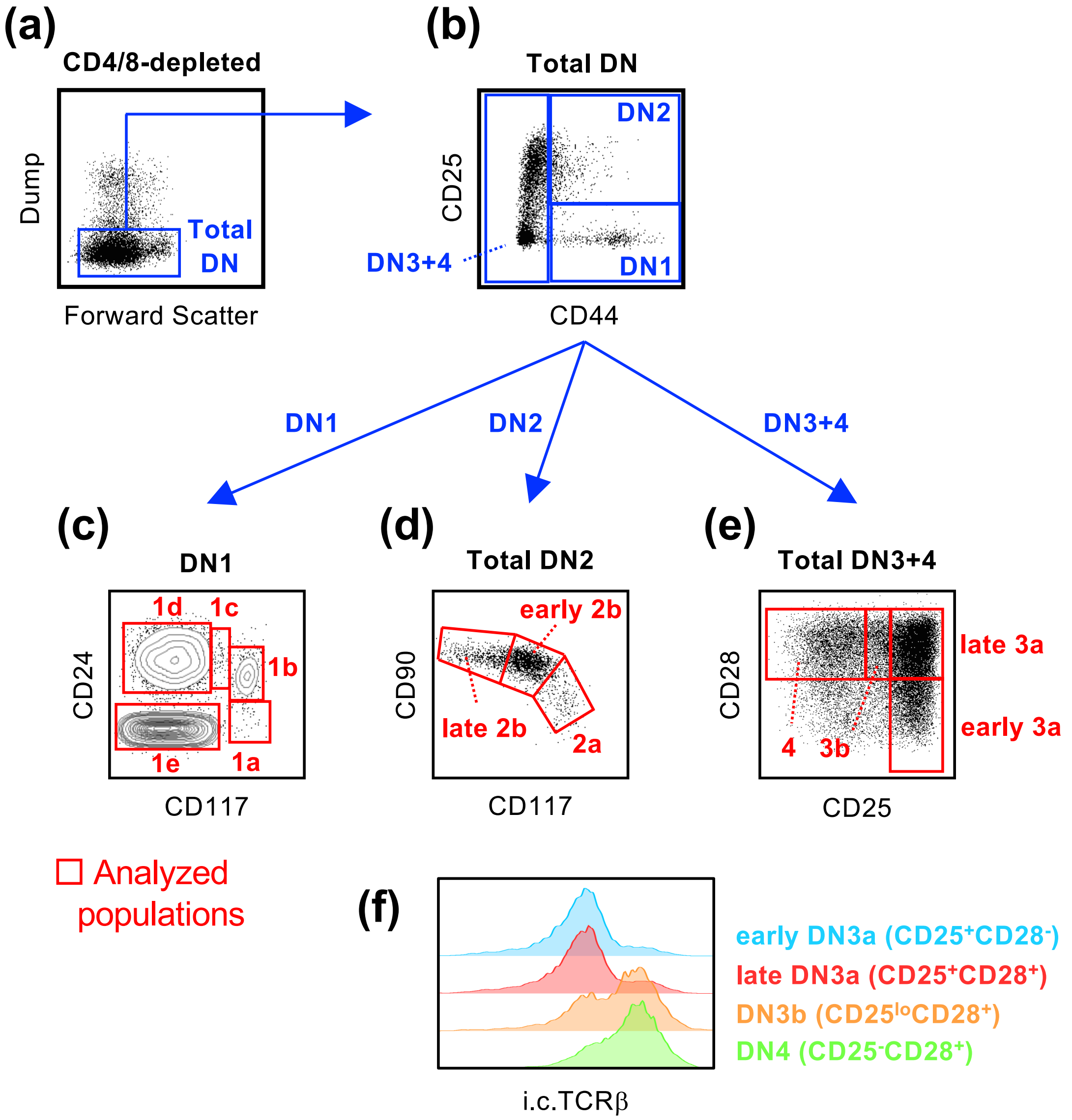

Supplementary figure 5: FACS gating strategy to identify DN thymocyte subpopulations. **(a)** Following MACS-depletion of CD4/8-expressing cells, total DN thymocytes were identified by gating out CD4/CD8/TCRβ/TCRγδ/CD11b/CD11c/NK1.1/B220-expressing cells. **(b)** The DN cells were then separated into DN1 to 4 by CD44 v CD25 expression. **(c)** DN1 ($CD44^{+}CD25^{-}$) cells were then subdivided into DN1a to DN1e by CD117 v CD24 expression. **(d)** DN2 ($CD44^{+}CD25^{+}$) cells were then subdivided into DN2a, early DN2b and late DN2b by CD117 v CD90 expression. **(e)** DN3+4 ($CD44^{-}CD25^{+/-}$) cells were then subdivided into early DN3a, late DN3a, DN3b and DN4 by CD25 v CD28 expression. **(f)** Confirmation that the $CD25^{lo}CD28^{+}$ gate within the DN3+4 ($CD44^{-}CD25^{+/}$) population corresponds to true DN3b cells with high intracellular (i.c.) TCRβ expression.

Supplementary table 1: Antibodies used for study

Antibody	Clone
Anti-mouse CD117	ACK2
Anti-mouse CD11b	M1/70
Anti-mouse CD11c	N418
Anti-mouse CD24	M1/69
Anti-mouse CD25	PC61.5
Anti-mouse CD28	37.51
Anti-mouse CD4	RM4-5
Anti-mouse CD44	IM7
Anti-mouse CD45R (B220)	RA3-6B2
Anti-mouse CD8α	53-6.7
Anti-mouse CD90.2	53-2.1
Anti-mouse TCRβ	H57-597
Anti-mouse TCRγδ	eBioGL3
Anti-NK1.1	PK136
